# Health Tract, No. 275

**Published:** 1867-02

**Authors:** 


### DENTISTRY.

Good teeth, good looks and good health are inseperable. Ill health destroys the teeth ; unless food is chewed well the horrors of a life-long dyspeptic are inevitable. The handsomest face in the world is marred, fatally marred by a snaggled tooth. The time to lay the foundation for a set of sound, solid teeth is when the child first begins to eat bread.—The finest set of teeth I ever saw in mortal man, induced me to stop the stranger and ask him if they were natural and how he accounted for their perfection of beauty ; he was forty-five years of age, not one missing—not one irregular—not one discolored, and so beautifully white that the sight was charming. He said he had thought on the subject a great deal, especially as all the younger members of the family had very poor teeth ; and he had settled it in his own mind that it was the secret of his father being so very poor when first married and for several years afterwards that, living in an out-of-a-way place they used a bread of corn or wheat or rye, as they could get it, rudely pounded into a very coarse meal. At the end of the first few years his father got a little ahead in the world and the younger children were all brought up as he was, except that they had the regular bread made of the common flour and meal ; hence he could come to no other conclusion than that the beauty of his teeth was owing to the qnality of bread eaten.

Scientific men within the last few years have come to a similar conclusion, and have solved the mystery with as much clearness, perhaps, as can be vouchsafed to questions of that kind.

Of the body of a tooth, seventy-one parts, nearly three-fourths are composed of lime, while of the enamel, upon the perfection of which depends the safety and durability of the teeth, ninety-four parts out of a hundred are lime. Hence the tooth is mainly made of lime. We get almost our entire supplies of lime for the teeth and bones from the bread we eat ; but observe, the bran, the outer covering of corn and wheat, is seperated from the flour and meal and thrown away ; but fine flour contains only thirty-five parts of elements of bone out of five hundred while bran contains one hundred and twenty five parts of the element of bone out of every five hundred. If, then, you want strong boned and perfect toothed children feed them on bread made from the whole product of the grain, from the time they begin to eat bread, beginning too, with the mother, to make assurance doubly sure, a year before they are born.

Many Dentists inculcate two most mischievous errors. Threads should never be drawn between the teeth. A permanent tooth ought never to be extracted to make room for others ; Nature knows what she is about ; every tooth is needed to develop the jaw, and that is of more importance than regularity. Soft brushes only should be used for the teeth and no wash except soap-suds twice a week, and every night and morning the following : Dissolve two ounces of borax in three pints of boiling water ; then add a teaspoonful of spirits of camphor ; keep it well bottled. A tablespoonful in as much warm water at a time. Or dip a brush in water and rub it on the teeth until the accumulation of saliva is sufficient.—This makes the softest, saftest and most cleansing tooth wash known.

